# Liquiritin Carbomer Gel Cold Paste Promotes Healing of Solar Dermatitis in Mice

**DOI:** 10.3390/ijms25073767

**Published:** 2024-03-28

**Authors:** Yanfang Huang, Sijia Li, Jinghua Pan, Congjing Song, Weiqiang Chen, Yun Zhang

**Affiliations:** 1 School of Nursing, Guangdong Pharmaceutical University, Guangzhou 510006, China; 2 School of Pharmacy, Guangdong Pharmaceutical University, Guangzhou 510006, China; 3 School of Basic Medical Sciences, Guangdong Pharmaceutical University, Guangzhou 510006, China

**Keywords:** solar dermatitis, liquiritin carbomer gel cold paste, anti-inflammatory, wound healing

## Abstract

Ultraviolet radiation (UVR) has various effects on human cells and tissues, which can lead to a variety of skin diseases and cause inconvenience to people’s lives. Among them, solar dermatitis is one of the important risk factors for malignant melanoma, so prevention and treatment of solar dermatitis is very necessary. Additionally, liquiritin (LQ) has anti-inflammatory effects. In this study, we aimed to evaluate the anti-inflammatory and pro-wound healing effects of liquiritin carbomer gel cold paste (LQ-CG-CP) in vitro and in vivo. The results of MTT experiments showed no cytotoxicity of LQ at concentrations of 40 μg/mL and below and cell damage at UVB irradiation doses above 60 mJ/cm^2^. Moreover, LQ can promote cell migration. ELISA results also showed that LQ inhibited the elevation of the inflammatory factors tumor necrosis factor-α (TNF-α), interleukin-1β (IL-1β), and interleukin-6 (IL-6) after UVB irradiation. In the mouse model of solar dermatitis, 2% LQ-CG-CP showed the best therapeutic efficacy for wound healing and relief of itching compared to MEIBAO moist burn moisturizer (MEBO). What is more, the results of skin histopathological examination show that LQ-CG-CP promotes re-epithelialization, shrinks wounds, and promotes collagen production, thus promoting wound healing. Simultaneously, LQ-CG-CP reduced TNF-α, IL-1β, and IL-6 expression. In addition, LQ-CG-CP was not observed to cause histopathological changes and blood biochemical abnormalities in mice. Overall, LQ-CG-CP has great potential for the treatment of solar dermatitis.

## 1. Introduction

Nowadays, UVR has become one of the most important factors affecting people’s lives due to its various effects on human skin tissue. Specifically, solar dermatitis, also known as sunburn, is an inflammation of the skin caused by exposure to excessive UVR in a sun-exposed environment, which usually leads to the appearance of erythematous pimples and blisters in sun-exposed areas, and self-consciousness of burning, itching, and pain [1]. The shoulders, neck, head, and face are the most common areas of sunburn, followed by the hands or arms and back [2]. The symptoms of solar dermatitis can bring inconvenience to patients’ lives. A study analyzing data from a national sample of U.S. hospital emergency department visits in 2013 estimated 33,826 sunburn-related visits and an estimated USD 11.2 million in costs associated with emergency department visits [3]. A history of solar dermatitis is an important risk factor for malignant melanoma [4]. Therefore, the prevention and treatment of solar dermatitis is essential.

UVR is categorized into three types based on their wavelengths: UVA (315–400 nm), UVB (280–315 nm), and UVC (100–280 nm); the atmosphere filters out most of the UVB and all of the UVC, so that the UVA and a portion of the UVB that reaches the Earth can cause damage to the human skin [5]. However, both act differently on the skin and only UVB induces characteristic epidermal sunburn damage [6]. UVB can induce an inflammatory response through several mechanisms [7,8]. In addition, UV can directly activate keratinocytes and other cells to release inflammatory mediators such as TNF-α, IL-1β, and IL-6 [9]. It has been shown that naringenin can inhibit skin edema induced by UVB irradiation, and also inhibit UVB irradiation-induced MMP-9 activity and the production of inflammatory factors to prevent UVB irradiation damage to mouse skin [10]. Verbenacea extract also had a protective effect on UVB-irradiated mice, inhibiting UVB-induced inflammatory responses as well as oxidative stress [11].

In recent years, the role of licorice flavonoids has attracted the attention of some researchers, and their potential medicinal value and application areas have been continuously studied, and their newly developed products have better application prospects [12,13]. LQ is a licorice flavonoid compound extracted from licorice [14]. Its anti-inflammatory effects have been demonstrated in studies of diseases such as rheumatoid arthritis and lipopolysaccharide-induced acute lung injury [15,16].

Currently, the main research focus is on the protective and preventive effects of solar dermatitis, while this paper focuses on the therapeutic perspective. The carbomer gel selected in this study can prolong the retention time of the drug on the skin surface, improve the efficiency of drug use, and, on this basis, when combined with a cold compress paste, can soothe the burning, itching, and other discomforts caused by solar dermatitis. Therefore, this study proposed to study the therapeutic effect of LQ-CG-CP in promoting wound healing of solar dermatitis in mice and explore the mechanism of its treatment of solar dermatitis, to provide a certain research basis for the subsequent development of the cold paste of solar dermatitis therapeutic gel, which has a certain clinical application prospect.

## 2. Results

### 2.1. Effect of LQ and UVB on Cell Proliferation Viability

**Figure 1:**
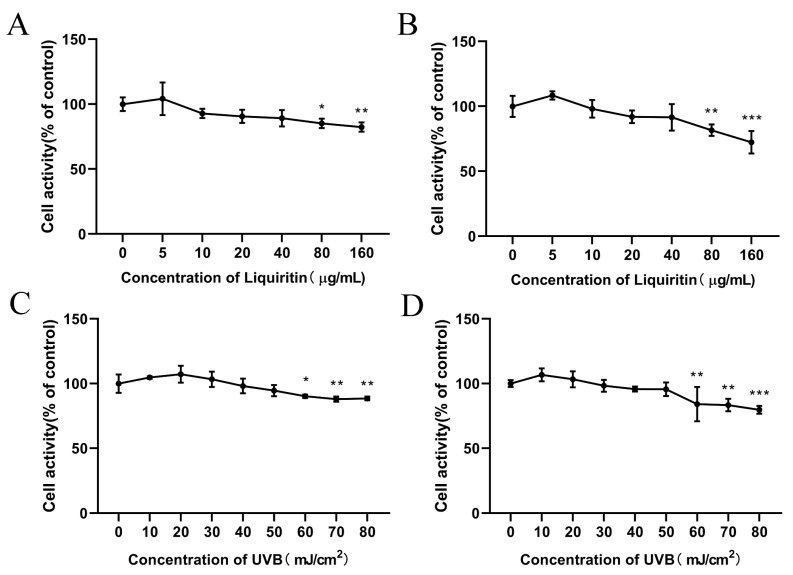
Effect of LQ and UVB on cell viability. (**A**) Effect of LQ on HaCaT cell viability; (**B**) Effect of LQ on JB6 cell viability; (**C**) Effect of UVB on HaCaT cell viability; and (**D**) Effect of UVB on JB6 cell viability. (ns > 0.05, * *p* < 0.05, ** *p* < 0.01, *** *p* < 0.001).

To confirm the concentration of LQ used and the UVB modeling dose, the MTT assay was used to detect the effects of LQ and UVB on HaCaT and JB6 cells. As shown in Figure 1A,B, the viability of HaCaT and JB6 cells was significantly decreased at concentrations of LQ of 80 μg/mL or more, indicating that high concentrations of LQ were significantly toxic to the cells (*p* < 0.05). Therefore, 40 μg/mL of LQ was used as the highest concentration for subsequent experiments. The survival rates of HaCaT and JB6 cells were significantly decreased (*p* < 0.05) when UVB was at a dose of 60 mJ/cm^2^ or above (Figure 1C,D). Compared with the blank control group, the cell survival rate in the 60 mJ/cm^2^ dose group was higher than that in the 70 and 80 mJ/cm^2^ dose groups, although it decreased; so, the 60 mJ/cm^2^ dose of UVB was the optimal dose for the cell model.

### 2.2. Effect of LQ on Cell Migration Capacity and UVB on Cell Secretion of Inflammatory Factors

**Figure 2:**
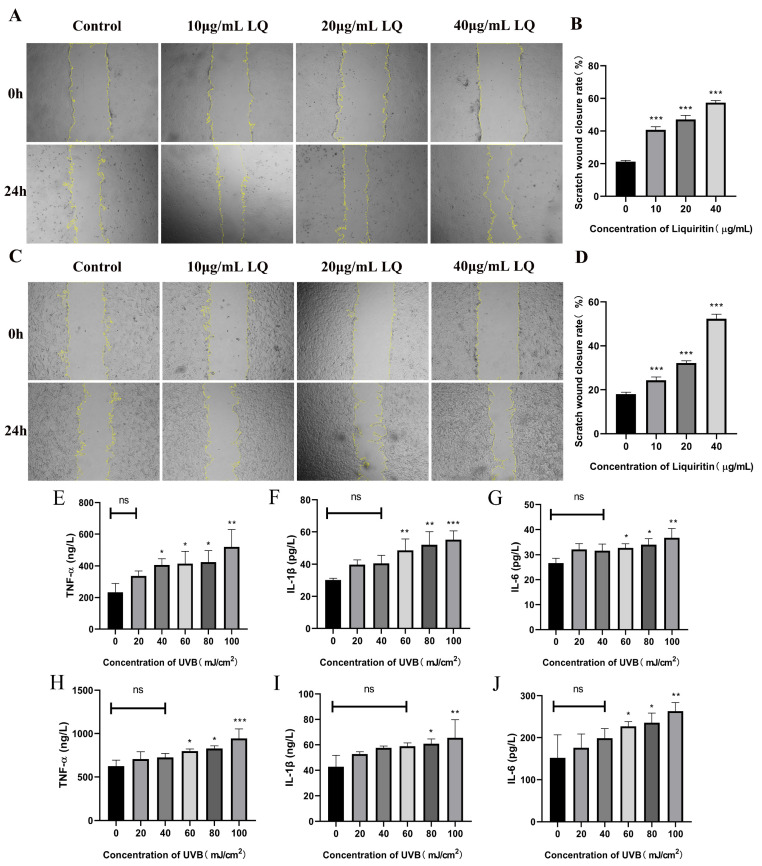
Effects of LQ and UVB on HaCaT and JB6 cells. (**A**) The change process of HaCaT cell scratch; (**B**) The scratch wound closure rate of HaCaT cells; (**C**) The change process of JB6 cell scratch; (**D**) The scratch wound closure rate of JB6 cells; (**E**–**G**) UVB-induced expression levels of inflammatory factors TNF-α, IL-1β, and IL-6 in HaCaT cells; and (**H**−**J**) UVB-induced expression levels of inflammatory factors TNF-α, IL-1β, and IL-6 in JB6 cells. (ns > 0.05, * *p* < 0.05, ** *p* < 0.01, *** *p* < 0.001, magnifications, ×40).

To assess whether LQ could promote cell migration, the cell scratch assay was used to detect the effects of different concentrations of LQ on the migration rate of HaCaT and JB6 cells. As shown in Figure 2A–D, LQ significantly increased HaCaT and JB6 cell scratch wound migration width (*p* < 0.05). It is thus clear that LQ promotes HaCaT and JB6 cell migration.

To determine the effect of UVB on cell secretion of inflammatory factors, the ELISA assay was used to detect the effects of different UVB irradiation doses on the expression levels of cellular inflammatory factors. As shown in Figure 2E–J, the higher the UVB modeling dose, the higher the levels of TNF-α, IL-1β, and IL-6.

### 2.3. Effect of LQ on Cell Secretion of Inflammatory Factors after UVB Irradiation

**Figure 3:**
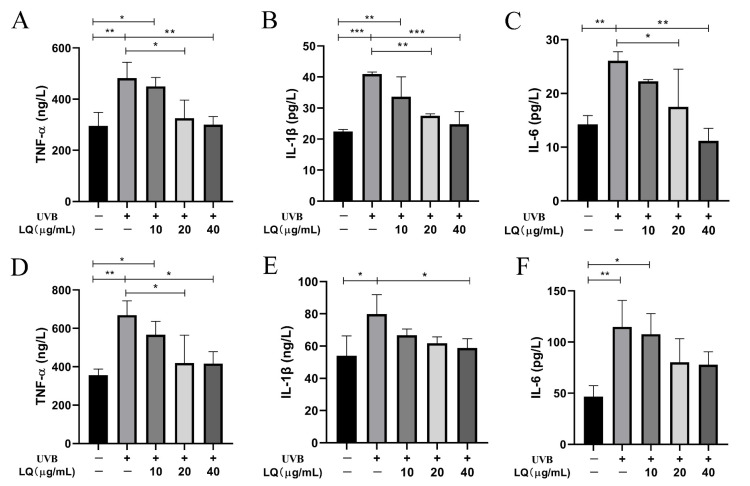
Effect of LQ on the expression levels of inflammatory factors in UVB-induced HaCaT and JB6 cells. (**A**–**C**) The levels of TNF-α, IL-1β, and IL-6 in HaCaT cells; and (**D**–**F**) The levels of TNF-α, IL-1β, and IL-6 in JB6 cells. (* *p* < 0.05, ** *p* < 0.01, *** *p* < 0.001; +: Positive, −: negative).

To determine whether LQ could inhibit the elevation of inflammatory factors in HaCaT and JB6 cells induced by UVB irradiation, ELISA experiments were used to detect the effects of different LQ concentrations on the expression levels of inflammatory factors in HaCaT and JB6 cells after UVB irradiation. As shown in Figure 3, the 60 mJ/cm^2^ UVB irradiation dose significantly increased the expression levels of the inflammatory factors TNF-α, IL-1β, and IL-6 in HaCaT and JB6 cells compared with the Control group (*p* < 0.01). In HaCaT cells, the administration of 20 μg/mL and 40 μg/mL LQ interventions significantly decreased the levels of TNF-α, IL-1β, and IL-6 (*p* < 0.05) and alleviated the inflammatory response in a dose-dependent manner (Figure 3A–C). In JB6 cells, the administration of 20 μg/mL and 40 μg/mL LQ interventions significantly decreased the level of TNF-α (*p* < 0.05) (Figure 3D); the administration of 40 μg/mL LQ interventions significantly decreased the level of IL-1β (*p* < 0.05) (Figure 3E); and the administration of LQ interventions failed to significantly decrease the level of IL-6, but it also followed an increase in dose and showed a decreasing trend (*p* > 0.05) (Figure 3F).

### 2.4. The Promoting Effect of LQ on Skin Wound Healing and Its Ability to Alleviate Itching Symptoms in Mice

**Figure 4:**
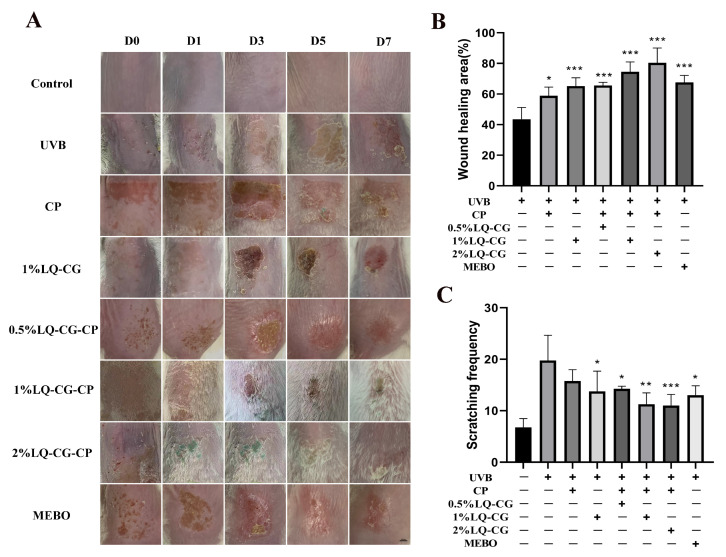
The characterization results of skin tissues during the treatment process. (**A**) Diagram of the skin wound healing process in each group of mice (scale bar, 100 μm); (**B**) Skin wound healing rate in each group of mice after 7 days of treatment; and (**C**) Pruritus situation in mice after 7 days of treatment. (* *p* < 0.05, ** *p* < 0.01, *** *p* < 0.001).

As shown in Figure 4A, which shows the process of skin wound changes in each group, the Control group is the normal skin of mice. Compared with the UVB group, the wound size was significantly smaller in the 1% LQ-CG-CP group and the 2% LQ-CG-CP group, which could also be found to be more effective than the application of CP and 1% LQ-CG alone. In addition, the effect of the positive drug MEBO was not as significant as that of LQ-CG-CP. As shown in Figure 4B, after 7 days of treatment, the average wound healing rates of mice in the UVB group, CP group, 1% LQ-CG group, 0.5% LQ-CG-CP group, 1% LQ-CG-CP group, 2% LQ-CG-CP group, and MEBO group were 43.5%, 58.86%, 65.20%, 65.64%, 74.50%, 80.34%, and 67.65%. Compared with the UVB group, the wound healing rates of mice in the CP, 1% LQ-CG, 0.5% LQ-CG-CP, 1% LQ-CG-CP, 2% LQ-CG-CP, and MEBO groups were significantly increased (*p* < 0.05), with the most significant healing effect in the 1% LQ-CG-CP and 2% LQ-CG-CP groups. What is more, mice with solar dermatitis develop itching symptoms, and the severity of itchy skin symptoms in mice was visually evaluated by observing the number of scratches. As shown in Figure 4C, compared with the UVB group, the itching number of mice in the CP group decreased slightly, while the itching behaviors of mice in the 1% LQ-CG group, the 0.5% LQ-CG-CP group, the 1% LQ-CG-CP group, the 2% LQ-CG-CP group, and the MEBO group could be significantly reduced (*p* < 0.05), with the greatest decrease in the number of scratches of mice in the 2% LQ-CG-CP group (*p* < 0.01), indicating the best effect.

### 2.5. Staining Results of Wound Skin Tissues

To study the process of LQ-CG-CP in promoting wound healing, mouse skin tissues were taken for HE and Masson staining to assess the rate of wound repair in mouse skin tissues after 7 days of administration.

**Figure 5:**
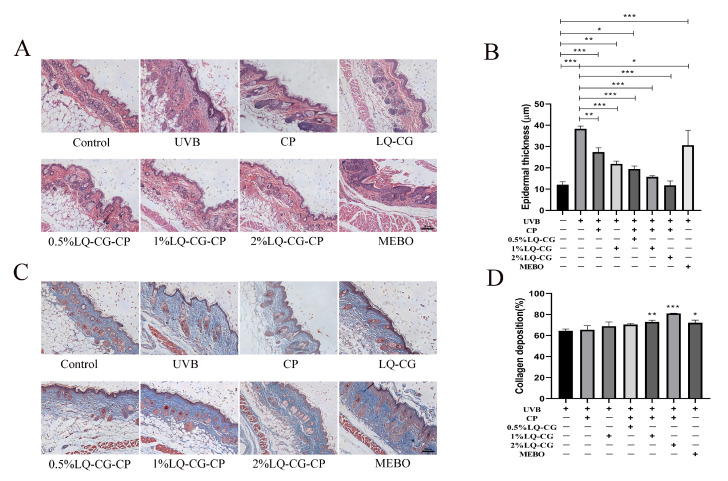
Results of HE and MT staining of skin wounds of mice in each group after 7 days of administration (100 μm). (**A**) HE staining; (**B**) Changes in epidermal thickness of the skin; (**C**) MT staining; and (**D**) Quantification of skin collagen deposition. (* *p* < 0.05, ** *p* < 0.01, *** *p* < 0.001, magnifications, ×100).

The HE staining results are shown in Figure 5A. Some areas in the UVB model group were not completely healed, did not possess complete epidermis, and still had inflammatory cell infiltration. There was a significant difference in epidermal thickness in the UVB, CP, 1% LQ-CG, 0.5% LQ-CG-CP, 1% LQ-CG-CP, and MEBO groups compared to the Control group (*p* < 0.05). Compared with the UVB group, the epidermal thickness of the CP, 1% LQ-CG, 0.5% LQ-CG-CP, 1% LQ-CG-CP, 2% LQ-CG-CP, and MEBO groups was significantly reduced (*p* < 0.05) (Figure 5B). In contrast, 2% LQ-CG-CP had formed a complete epidermis, which was close to the skin histological structure of the blank control group. The epidermis of the MEBO group was thickened, the epidermis was still not completely healed, and the stratum corneum was disorganized.

The collagen fibers of the skin tissue were blue after MT staining. As shown in Figure 5C, which shows the collagen deposition in each subgroup after 7 days of treatment, compared with the UVB and CP groups, the collagen fibers in the 1% LQ-CG-CP and 2% LQ-CG-CP groups were in a tightly ordered arrangement, with smaller epithelial tissue gaps, which was similar to the normal skin tissue structure in the Control group. Although a large number of collagen fibers were also formed in the MEBO group, their arrangement and distribution were more disorderly. In Figure 5D, it is shown that collagen deposition was significantly increased in the 1% LQ-CG-CP, 2% LQ-CG-CP, and MEBO groups compared to the UVB group (*p* < 0.05).

### 2.6. Effect of LQ-CG-CP on Inflammatory Factors in a Mouse Model of Solar Dermatitis

**Figure 6:**
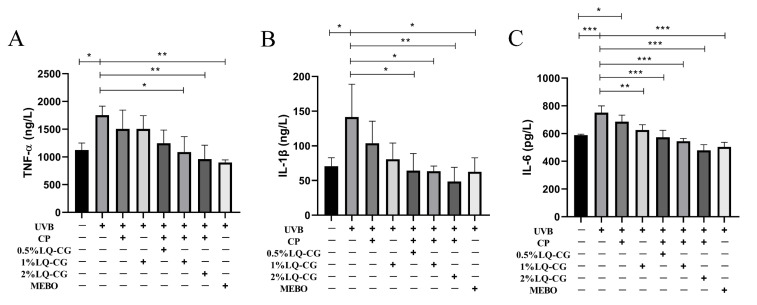
Levels of inflammatory factors in mice of each group after 7 days of administration. (**A**) Levels of TNF-α in mice of each group; (**B**) Levels of IL-1β in mice of each group; and (**C**) Levels of IL-6 in mice of each group. (* *p* < 0.05, ** *p* < 0.01, *** *p* < 0.001).

ELISA experiments were used to detect the effects of different LQ concentrations on the expression levels of inflammatory factors in a mouse model of solar dermatitis. As shown in Figure 6, the expression levels of inflammatory factors TNF-α, IL-1β, and IL-6 were significantly increased in the UVB modeling group of mice compared with the blank control group (*p* < 0.05). After 7 days of administration, the CP group could not significantly decrease the expression levels of TNF-α, IL-1β, and IL-6, while the administration groups showed a significant decrease (*p* < 0.05) in the expression levels of TNF-α, IL-1β, and IL-6 and alleviated the inflammatory response in a dose-dependent manner. In addition, the expression levels of IL-1β and IL-6 were lower in the 2% LQ-CG-CP group than in the positive drug MEBO group.

### 2.7. Biosafety Research

#### 2.7.1. Histopathologic Results of Mouse Viscera

**Figure 7:**
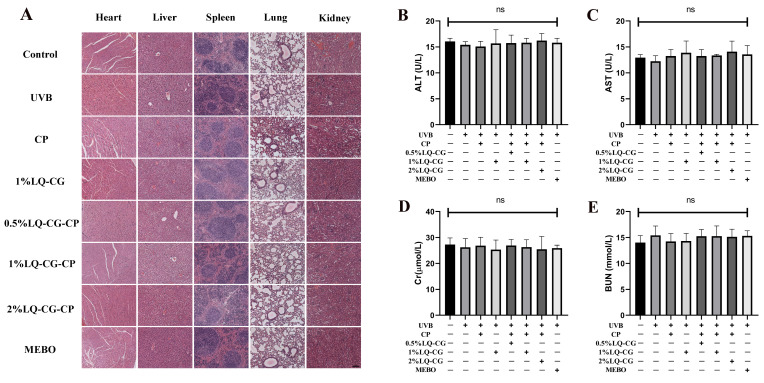
Biosafety assay. (**A**) HE staining results of heart, liver, spleen, lung, and kidney in each group after 7 days of treatment (100 μm); and (**B**–**E**) Blood biochemical indices (ALT, AST, Cr, and BUN) levels of hepatic and renal functions of mice in each group at the end of treatment. (ns > 0.05, magnifications, ×100).

After the mice in each group were given the relevant treatments, HE staining was performed on the major organs of the mice to assess the histopathological changes in each group. There were no histopathological changes in the heart, liver, spleen, lungs, and kidneys of mice in all groups compared with the Control group (Figure 7A).

#### 2.7.2. Blood Biochemical Indicators

The safety of LQ-CG-CP was further evaluated by measuring blood biochemical indices. There were no statistically significant differences in alanine aminotransferase (ALT), alanine transaminase (AST), serum creatinine (CREA), and blood urea nitrogen (BUN) in each group compared to the Control group (Figure 7B–E).

## 3. Discussion

This study aimed to investigate whether LQ-CG-CP significantly promotes wound healing in solar dermatitis mice. The results showed that LQ-CG-CP significantly accelerated wound healing.

Currently, sunburn dermatitis is common. In the 2005, 2010, and 2025 National Health Interview Surveys, the estimated percentage prevalence of sunburn (experiencing ≥1 sunburn in the past 12 months) among U.S. adults was 34.2%, 37.1%, and 34.1%, respectively [17]. Teenagers are more likely to experience sunburn. According to an Irish research study, 74% of school-age children (10–17 years) experienced sunburn in 2017, with 10% reporting ≥5 sunburns [18]. Ultraviolet radiation exposure is an important risk factor for skin inflammation and skin cancer, while ultraviolet overexposure and a history of associated solar dermatitis are important risk factors for skin cancer [19]. That is why the number of hospital outpatient emergency room visits to treat sunburns has increased as knowledge about the subject has become more widespread. However, there are also hospitalized sunburn patients; 19% of sunburn patients in the Australian and New Zealand study underwent burn wound management surgery, and 4% of these patients were admitted to the intensive care unit during their hospitalization [20]. Therefore, it is important to actively prevent or promptly treat solar dermatitis, as this can increase the risk of developing skin cancer.

Currently, LQ is widely used in the study of various diseases. In the present study, LQ was not significantly toxic to cells at concentrations of 5, 10, 20, and 40 μg/mL, and even promoted cell proliferation at 5 μg/mL (Figure 1A,B).

The skin wounding process is extremely complex and consists of three main processes: inflammation, new tissue formation, and remodeling. A series of reactions such as hemostasis, inflammation, angiogenesis, growth, re-epithelialization, and tissue remodeling occur sequentially and overlappingly during these three phases [21]. One of the main hallmarks of wound healing is wound reduction [22]. During wound healing, fibroblasts migrate toward the center of the wound and transform into α-SMA-positive myofibroblasts [22]. Fibroblasts of different origins are remarkably heterogeneous. Consequently, this leads to the emergence of differentially expressed genes, including extracellular matrix (ECM) synthesis, proliferation, and migration, all of which are closely related to wound healing [23]. The outermost layer of the skin is the epidermis, which is composed primarily of Keratinocytes. Fibroblasts and keratinocytes interact with each other, with fibroblasts producing signaling factors that promote the proliferation and migration of keratinocytes [24]. At present, a rapid, practical, direct, and reproducible in vitro artificial wound assay exists to determine the effect of drugs on cell migration. The wound healing assay, also known as the “cell scratch assay”, is a simple, versatile, and low-cost method to study the collective cell migration and wound healing capacity [25]. In addition, wound closure from the cell scratch assay can also be used to assess wound re-epithelialization [26]. The results showed that LQ promoted cell migration and reduced the scratch wound distance, especially the fastest migration of group 40 μg/mL (Figure 2A–D). It demonstrated that LQ has the ability to promote wound reduction and accelerate the wound-healing process.

UVB induces the release of TNF-α, IL-1β, and IL-6, and its secretion is influenced by the irradiation dose (Figure 2E–J). The balance of cytokines in the wound determines the optimal state of wound healing. For example, TNF-α can inhibit the differentiation of myofibroblasts, leading to prolonged inflammation and delaying wound healing to a certain extent [27]. Therefore, it is necessary to appropriately inhibit inflammatory factors. The results of this study show that LQ can effectively inhibit inflammatory factors, alleviate UVB-induced inflammatory response, and its effect is dose-dependent (Figure 3).

To investigate the effect of LQ-CG-CP on mice with solar dermatitis, we also conducted in vivo studies. The successful mouse model of solar dermatitis was constructed as follows: the skin appeared red and swollen at the end of modeling, edema appeared after 24 h, and the skin was edematous and broke down after 48 h.

Above all, wound healing was determined by the process of wound area change and wound healing rate. Macroscopically, the rate of wound reduction was faster in the LQ-CG-CP group as the duration of administration increased (Figure 4A). From the wound healing rate data, it can be concluded that the 1% LQ-CG-CP and 2% LQ-CG-CP groups had a higher healing rate than the MEBO group (Figure 4B). Surprisingly, after 7 d of treatment, the 2% LQ-CG-CP group had the best wound healing, with a complete epidermis having formed in the wound area.

To further investigate the effect of LQ-CG-CP on wound healing, mouse skin tissues were taken for histological examination. Wound healing is closely related to granulation tissue formation, re-epithelialization, and wound reduction. Tissue damage repair begins with granulation tissue formation [28]. During the wound healing phase, granulation tissue formation occurs concurrently with re-epithelialization. Keratinocytes are the protagonists of wound healing re-epithelialization, and they can repair epidermal barrier function through proliferation and migration. After re-epithelialization occurs, the wound shrinks and produces a new epidermis. The HE results showed that LQ-CG-CP promoted re-epithelialization of skin tissues; 2% of the LQ-CG-CP group formed a complete epidermis, which was similar to the normal epidermal morphology, while the MBEO group had a disorganized epidermal layer (Figure 5A). Subsequently, collagen synthesis in vivo was also assessed in this study by MT staining. Collagen is one of the primary ECM components in the skin and is secreted primarily by fibroblasts [29]. During the proliferative phase of wound healing, α-SMA-positive myofibroblasts synthesize specific ECM molecules that promote contractile remodeling of granulation tissue and facilitate wound contraction [30]. The results of MT experiments showed that LQ-CG-CP increased collagen expression and promoted collagen deposition, with the 2% LQ-CG-CP group showing the highest collagen deposition (Figure 5C,D). This demonstrated the good crude wound healing ability of LQ-CG-CP.

UVB promotes the production and release of inflammatory factors (TNF-α, IL-1β, and IL-6) to induce an inflammatory response. TNF-α, IL-1β, and IL-6 affect the proliferation and differentiation of keratinocytes as well as ECM formation [31]. While prolonged inflammation can prolong wound healing, it can also lead to the development of keloids or hyperplastic scars [32,33]. In addition, both the initiating signals for transcription of immature IL-1β and the generation of danger signals for the release of mature IL-1β lead to a macrophage-induced inflammatory response that dysregulates wound healing [34]. The results showed that LQ-CG-CP reduced the inflammatory response and inhibited the side effects associated with long-term inflammation (Figure 6). Moreover, LQ-CG-CP has been shown to have no side effects on mice in biosafety tests (Figure 7).

Sunburned skin, in addition to inflammation and skin damage, is characterized by intense itching, and the itching associated with sunburn is known as the “Hell’s Itch” [35]. This itching seriously affects the quality of life and must be effectively intervened. Cold compresses or cold showers can relieve itching [36]. This study also combined cold therapy for administration to reduce discomfort at the skin lesions. The decrease in the number of scratches of mice in the CP group compared with the UVB group, as well as the fact that the number of scratches in the 1% LQ-CG-CP group was less than that in the 1% LQ-CG group, could indicate that cold compresses could alleviate the itching symptom of mice (Figure 4C). However, the mechanism of UVB-induced itching is not fully understood. It has been studied that UVB irradiation induces pruritus in mice by promoting TRPV1 channel function in dorsal root ganglion neurons [37]. Future studies could delve deeper into the mechanisms of sunburn-related itching and the effects that interventions have on the mechanisms.

## 4. Materials and Methods

### 4.1. Cell Culture and Treatment

The human epidermal keratinocyte (HaCaT) cell line was taken from Suyan Biotechnology (Guangzhou, China), and the mouse epidermal JB6 cell line was obtained from Yiyou Biotechnology (Guangzhou, China). HaCaT cells were cultured in Dulbecco’s modified Eagle’s medium (DMEM, GIBCO, Grand Island, NE, USA) containing 10% fetal bovine serum (FBS, BIOIND, Kibbutz Beit Haemek, Israel) and antibiotics (100 U/mL penicillin and 100 U/mL streptomycin, GIBCO, Grand Island, NE, USA), while JB6 cells were cultured in Roswell Park Memorial Institute (RPMI) 1640 (GIBCO, Grand Island, NE, USA) containing 10% FBS and antibiotics (100 U/mL penicillin and 100 U/mL streptomycin, GIBCO, Grand Island, NE, USA). These cells were cultured at 37 °C with a 5% CO_2_ incubator.

LQ (Purity > 98%, CAS: 551-15-5) was purchased from Chengdu nakeli Biotechnology Co., Ltd. (Chengdu, China). The structural formula of LQ is provided in the Supplementary Materials (Figure S1). LQ was dissolved in dimethyl sulfoxide (DMSO, Sigma, St. Louis, MO, USA). The final concentration of DMSO was 0.1%, which had no significant effect on cell viability, as detailed in Supplementary Figure S2.

A UVB light source (Guanhongrui, Shenzhen, China) was used in this study, providing UV light in the range of 280–320 nm, with its spectral energy mainly concentrated at 308 nm. At the distance of 5 cm, the average UVB irradiation intensity was 3 W/m^2^.

### 4.2. Cell Viability Assay

The MTT assay was used to test cell viability. The cells were digested down with trypsin and prepared into cell suspensions. After the cells were counted, a cell suspension was prepared at a concentration of 4 × 10^4^ cells per well, and then 100 μL of each well was seeded in a 96-well plate. After 24 h incubation, the old medium was discarded and fresh medium was added, followed by UVB at different irradiation doses (0, 10, 20, 30, 40, 50, 60, 70, 80 mJ/cm^2^); or cells were cultured with different concentrations of LQ (0, 10, 20, 40, 80, 160 μg/mL) for 24 h. Then, cells were incubated for 4 h by adding 10 μL of MTT (Rsbio, Shanghai, China) per well. After that, the MTT mixture was discarded, and 100 μL of DMSO was added to each well, and the plate was shaken on a shaker for 10 min. Later, the absorbance at 490 nm was measured for each group using the Microplate Reader (Biotek, Winooski, VT, USA).

### 4.3. Cell Migration Assay

HaCaT cells and JB6 cells (5 × 10^5^ cells/well) were seeded in a six-well plate for the wound healing assay. When the cells were cultured to 90% confluence, the cells were pretreated with a low dose of mitomycin c (5 μg/mL) for 2 h. After removing the mitomycin c-containing medium and washing it once with PBS, a straight line was drawn vertically with a 200 uL pipette tipper well. Dropped cells were washed out with phosphate-buffered saline (PBS, Meilunbio, Dalian, China). And then, DMEM containing different concentrations of LQ (10, 20, 40 μg/mL) was added to each well. Groups of scratched areas were photographed at 0 h and 24 h using a microscope (Olympus, Tokyo, Japan). The scratch wound closure rate was determined by the following equation: Scratch wound closure rate = (Scratch area at 0 h − Scratch area at 24 h)/Scratch area at 0 h × 100%.

### 4.4. Preparation of LQ-CG-CP

Carbomer 940 gel does not cause skin irritation and is the vehicle for the drug application in this article. First, 1 g of carbomer 940 powder (Meilunbio, Dalian, China) was mixed with 100 mL of ultrapure water and stirred for 5 min, and then the pH was adjusted to 7.0 with triethanolamine (Macklin, Shanghai, China). Subsequently, three different concentrations of LQ were added to formulate a 1% carbomer gel containing 0.5%, 1%, and 2% (*w*/*v*) LQ. The previous step requires stirring at 25 °C until a homogeneous and good-quality gel is formed [38]. What is more, cold paste (Haixu, Rizhao, China) is pre-made. LQ-CG-CP was prepared by uniformly applying LQ-containing carbomer gel to the cold paste. Its relevant characterization and in vitro drug release assays can be found in Figures S3 and S4 of the Supplementary Materials.

### 4.5. Establishment and Treatment of Mouse Solar Dermatitis Model

All experimental animal procedures were carried out following the Guidelines for the Care and Use of Laboratory Animals at Guangdong Pharmaceutical University and approved by the Animal Ethics Committee (No. gdpulacspf2022092). 8 w BALB/c female mice were purchased from Guangdong Laboratory Animal Center. The experimental animals were housed at a temperature of 23–25 °C and humidity of (50 ± 15)%, kept in alternating light and dark for 12 h. Feed, drinking water, and bedding were changed regularly. Mice were first adapted to rearing for 1 w, and dorsal dehairing was performed the night before modeling. Except for the control group, the rest of the mice were exposed to UVB light irradiation. The UVB irradiation dose is 5 J/cm^2^ (About 4.6 h). After 48 h, mice were divided into the UVB group, 1% LQ-CG group, 0.5% LQ-CG-CP group, 1% LQ-CG-CP group, 2% LQ-CG-CP group, and positive drug group (MEBO, Shantou, China). According to the grouping, the corresponding dose was given externally on the skin of mice once a day for 7 days. At the same time, the skin of the mice was photographed and recorded at the same height every day. The size of skin wounds in mice was measured using Image J software (Version 1.80; NIH, Bethesda, MD, USA), and wound healing rates were calculated. Moreover, at the end of the treatment, the mice were placed in transparent boxes and videotaped for one hour to record the scratching frequency of the mice. After 7 d of treatment, mice were anesthetized with isoflurane and then sacrificed, and skin tissues and major organ tissues were removed.

### 4.6. Enzyme-Linked Immunosorbent Assay (ELISA)

ELISA kits (Jiangsu Meimian Industrial Co., Ltd., Yancheng, China) were used to detect the secretory levels of TNF-α, IL-1β, and IL-6. Supernatants were collected from JB6 and HaCaT cells after 24 h of UVB irradiation or 24 h of UVB irradiation followed by 24 h of incubation with LQ. Serum was extracted from mice with a model of solar dermatitis given LQ for 7 d. According to the manufacturer’s instructions, supernatants and serum were used to detect the expression of relevant indicators.

### 4.7. Histopathological Examination

After the skin tissues were taken, they were fixed in 4% paraformaldehyde (Biosharp, Bengbu, China) for 24 h, embedded and deparaffinized, and then stained with hematoxylin and eosin (HE). In addition, skin tissues were stained for Masson’s trichrome (MT). Pathologic changes in skin tissue were observed through the microscope. Later, epidermal thickness and collagen deposition quantification (Collagen deposition quantification = Collagen area/Total organizational area × 100%) were measured using Image J software (Version 1.80; NIH, Bethesda, MD, USA).

### 4.8. Biosafety Validation

Following the steps above, the major organs of mice were taken for histopathological analysis. In addition, serum samples from mice were used for biochemical assays, which contained four indicators: ALT, AST, Cr, and BUN. The levels of ALT, AST, Cr, and BUN were measured according to the kit manufacturer’s instructions (Nanjing Jiancheng, Nanjing, China).

### 4.9. Statistical Analysis

Results were shown as the mean ± standard deviation (SD). Statistical significance was assessed by *t*-test or one-way ANOVA using Graph Pad Prism 8.0 software. Comparisons of more than two groups were corrected using the Bonferroni test. *p* < 0.05 indicates statistical significance. All experiments were repeated three times.

## 5. Conclusions

In this study, we evaluated the therapeutic efficacy of LQ-CG-CP in solar dermatitis. LQ-CG-CP inhibits inflammatory reactions, reduces itching symptoms, promotes collagen production, and promotes wound healing. In conclusion, LQ-CG-CP shows great potential in the treatment of solar dermatitis and deserves further research.

## Data Availability

The data that support the findings of this study are available from the corresponding author upon reasonable request.

## Supplementary Materials

The following supporting information can be downloaded at: https://www.mdpi.com/article/10.3390/ijms25073767/s1.
