# Supplementary material for: Liquiritin Carbomer Gel Cold Paste Promotes Healing of Solar Dermatitis in Mice
Source: Int J Mol Sci. 2024 Mar 28;25(7):3767. doi: 10.3390/ijms25073767 (PMC11011678; doi:10.3390/ijms25073767)
Supplement: Supplementary file 1 [file ijms-25-03767-s001.zip › ijms-2844462-supplementary.pdf]

Supplementary Materials

1. The structural formula of liquiritin

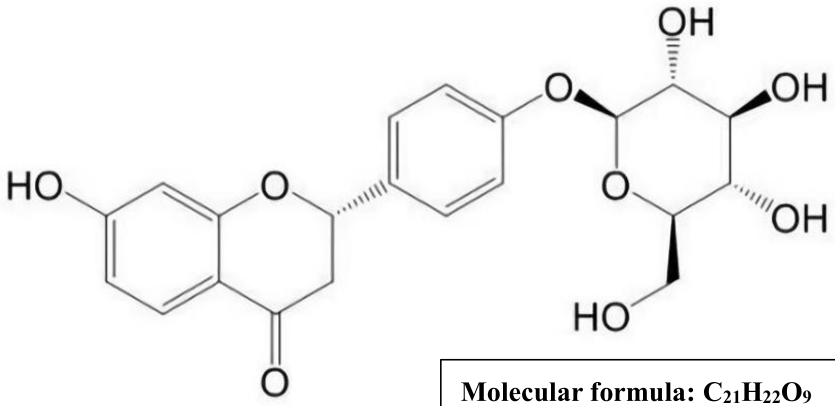

Molecular formula: $C_{21}H_{22}O_9$
Molecular weight: 418.39

Figure S1. The structural formula of liquiritin

2. Effect of 0.1% DMSO on cell viability

To confirm whether 0.1% DMSO would have an effect on the cells, MTT assay was used to detect cell viability. As the result in Figure S2 showed, 0.1% DMSO had no effect on HaCaT and JB6 cells.

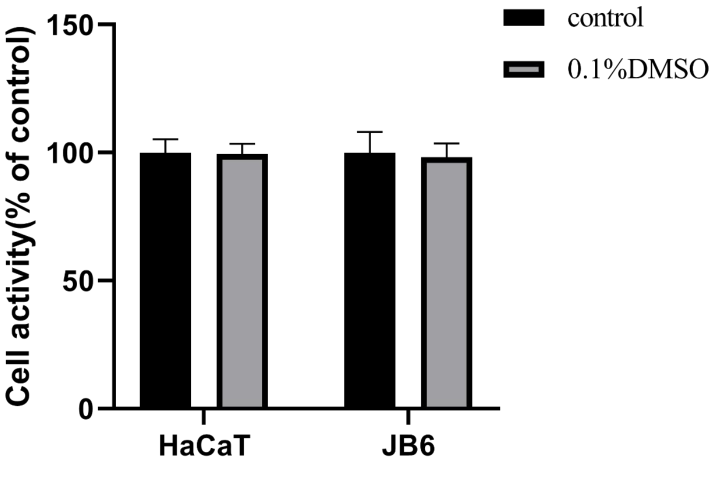

Figure S2. Effect of 0.1% DMSO on cell viability

3. Characterization

After preparation of 0.5% LQ-CG,1% LQ-CG and 2% LQ-CG, the samples were lyophilized in a lyophilizer. Subsequently, gold was sprayed on the surface of the samples, and SEM images were scanned and recorded using a Hitachi SU8100 SEM (Tokyo, Japan) to observe the microstructure of LQ-CG.

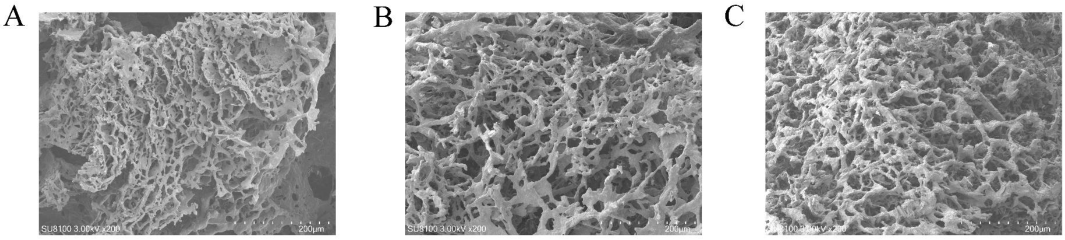

Figure S3. SEM images of LQ-CG. (A) 0.5% LQ-CG; (B) 1% LQ-CG; and (C) 2% LQ-CG

In order to observe the microstructure of LQ-CG, the surface of the hydrogel was observed morphologically by SEM. SEM images show that the LQ-CG pore sizes are similar in size and the pore distribution is uniform. Having a large number of pores and a well-developed three-dimensional structure helps with cell infiltration and promotes the formation of new tissues, which is beneficial for wound healing.

4. The study of drug release in vitro

In order to study the release of LQ from LQ-CG, a standard absorbance curve for LQ was established. The calibration curve was y = 0.0011x + 0.717, R^2= 0.99. The release of LQ-CG in a simulated in vitro environment (pH 7.4 PBS) was determined. Firstly, 1 mL of LQ-CG was prepared, 5 ml of PBS was added and placed at 37°C. At a specific time, 1 ml of PBS was removed and 1 ml of PBS was added and the absorbance of the solution was measured at 276 nm. The cumulative release of drug from LQ-CG was calculated according to the following equation. E represents the cumulative percent release of LQ-CG. V_E is the volume of buffer taken out, V_0 is the volume of PBS added initially, C_i and C_n represent the concentration of the drug at the i-th and n-th hour, and M_0 represents the mass of the drug in the LQ-CG at the time of initial.

$$E\ (\%) = \left(V_E \sum_1^{n-1} C_i + V_0 C_n \right) / m_o \times 100\%$$

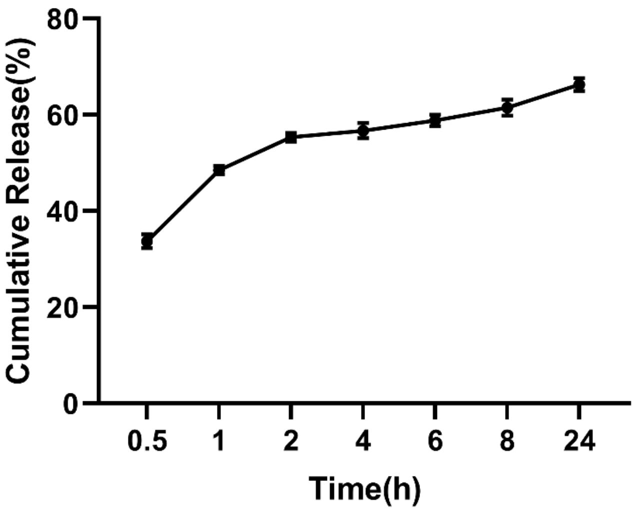

Figure S4. In vitro drug release of LQ-CG

The results of in vitro drug release showed that the total cumulative release of LQ reached 55.34% at 4 h, after which the release rate slowed down to a cumulative release of 66.3% at 24 h.
